# Cardiac Contusion Complicated by Heart Failure in a Young Athlete

**DOI:** 10.1016/j.jaccas.2022.07.008

**Published:** 2022-09-07

**Authors:** Krishna Pabba, R. Jay Widmer, Vinh Nguyen, Matthew W. Martinez

**Affiliations:** a Department of Cardiovascular Medicine, Baylor Scott & White Medical Center, Temple, Texas, USA; b Department of Cardiovascular Medicine, Morristown Medical Center, Atlantic Health System, Morristown, New Jersey, USA

**Keywords:** cardiac contusion, competitive athletics, heart failure, multimodality imaging, BCI, blunt chest injury, LGE, late gadolinium enhancement

## Abstract

Chest trauma is a relatively common injury in athletes. Here, we report a case of a cardiac contusion in a football player that led to hemodynamically significant low-output state. Early invasive management was critical in treatment with imaging playing an important role in diagnosis. (**Level of Difficulty: Advanced.**)

## Central Illustration

**Figure:**
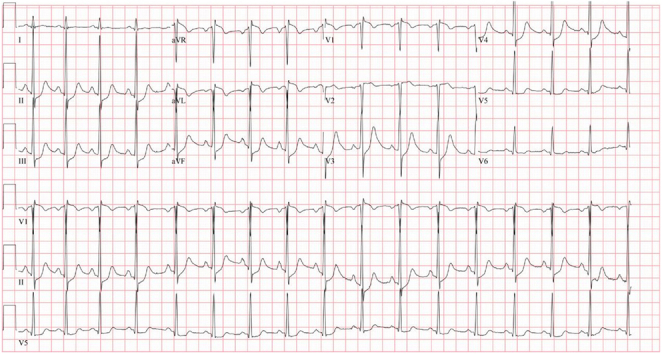

## History of Presentation

A 23-year-old linebacker presented to the emergency department as a trauma activation due to a helmet impact to the chest while playing college American football. He did not have loss of consciousness but expressed that he felt “the wind knocked out of him.” He left the game and shortly after started to have chest pain, shortness of breath, and intractable vomiting. His initial blood pressure was 158/100 mm Hg, heart rate 99 beats/min, respiratory rate 22 breaths/min, and oxygen saturation 78%. Physical examination was notable for diffuse rales.

**Learning Objectives**

• To suspect BCI in athletes with chest trauma and ECG abnormalities or elevated troponin.
• To use multimodality imaging to accurately diagnose and treat BCI.

## Past Medical History

There was no significant medical history.

## Differential diagnosis

Pericardial tamponade, coronary artery dissection, cardiac contusion, and valvular injury were considered.

## Investigations

**Figure 1:**
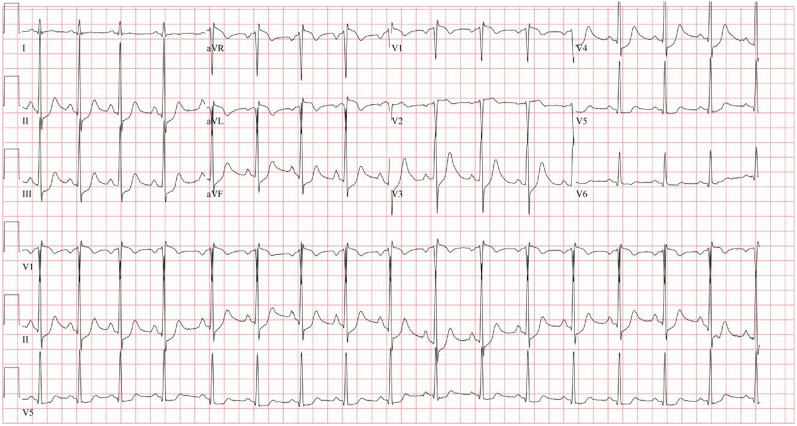
Initial Electrocardiogram

The patient had worsening hypoxic respiratory failure necessitating intubation. A chest x-ray revealed diffuse bilateral opacities consistent with pulmonary edema. Electrocardiogram (ECG) showed diffuse ST-segment depressions with ST-segment elevation in aVL, aVR, and V_1_, which were concerning for injury vs global ischemia (Figure 1). Initial troponin-I was 3 ng/mL (normal range 0 to 0.05 ng/mL) and peaked at 39 ng/mL. Computed tomography (CT) of the chest/abdomen/pelvis showed pulmonary edema and right adrenal hemorrhage. Urgent bedside echocardiography did not show tamponade. However, it did reveal a dilated left ventricle with global hypokinesis with an estimated ejection fraction of 20%.

**Figure 2:**
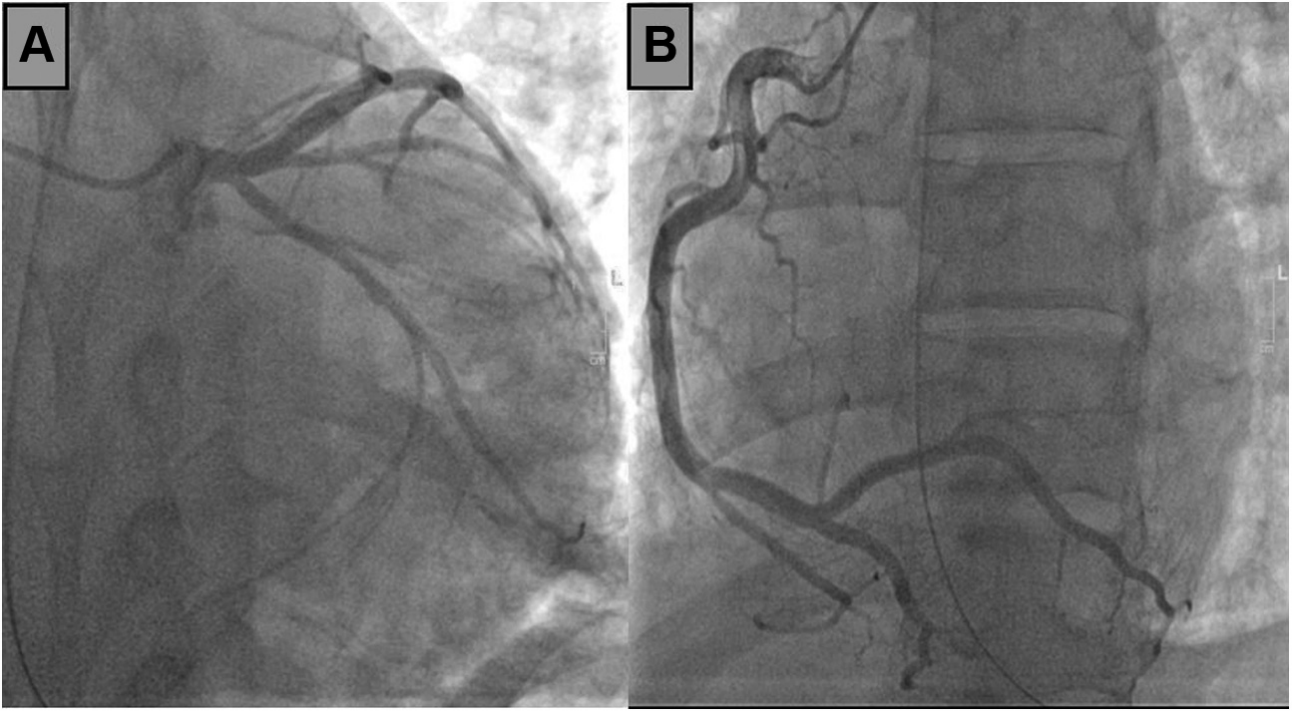
Coronary Angiography **(A)** Left coronary artery. **(B)** Right coronary artery.

After initial stabilization in the emergency department, cardiac catheterization was performed which did not show angiographic evidence of significant coronary disease or dissection (Figure 2). Right heart catheterization was significant for a cardiac index 2.02 L/min/m^2^, pulmonary capillary wedge pressure 31 mm Hg, systemic vascular resistance 1,598 mm Hg⋅min⋅mL^-1^**,** cardiac power output 0.92 W, right atrial pressure 10 mm Hg, and pulmonary artery pulsatility index 2.3.

## Management

The patient was brought to the cardiac intensive care unit and was started on a nitroglycerin drip, captopril for afterload reduction, and furosemide. He was able to be extubated within 24 hours and goal directed medical therapy was titrated.

**Figure 3:**
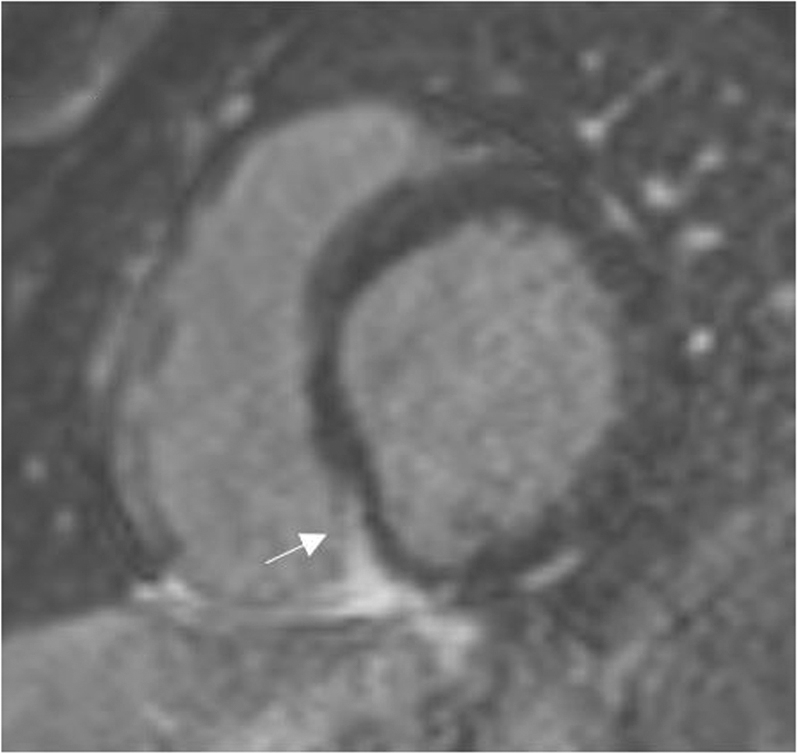
Late Gadolinium Enhancement **Arrow** indicates late gadolinium enhancement at the basal inferoseptal segment of the right ventricular septum.

**Figure 4:**
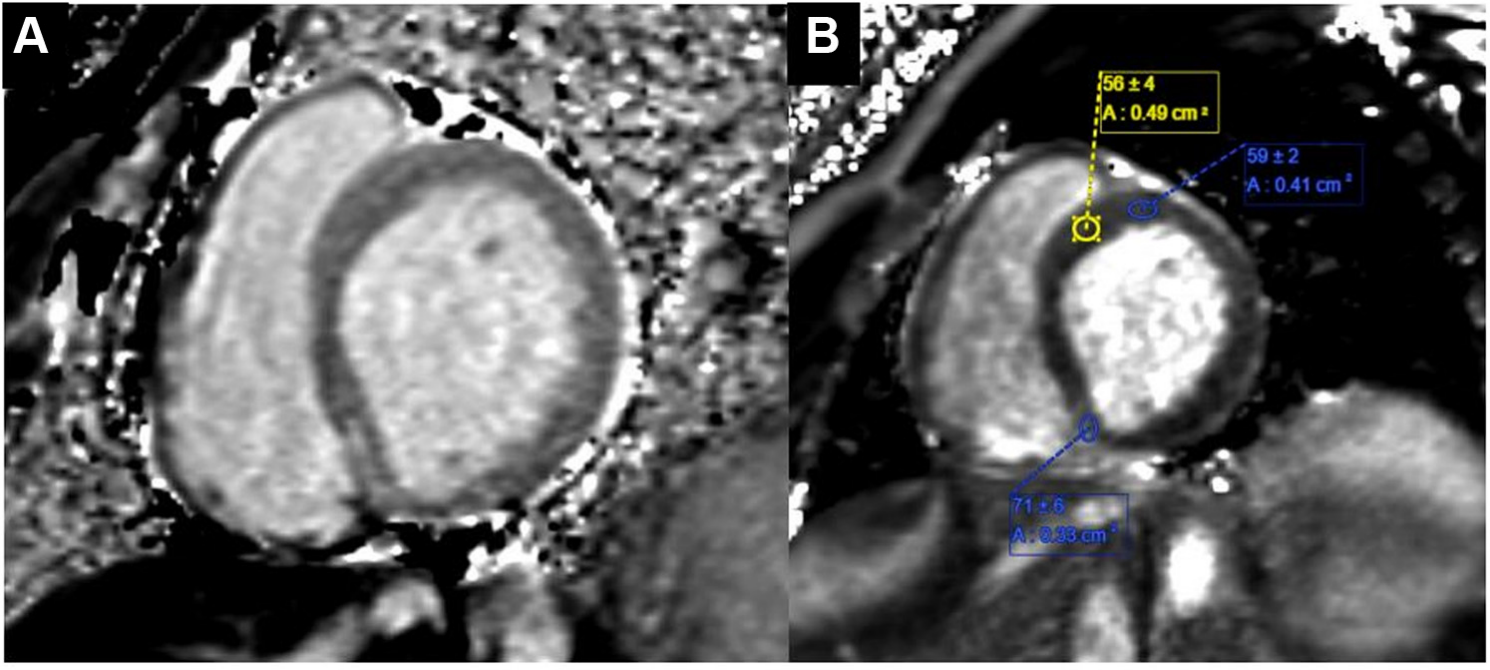
Increased T1 and T2 Mapping **(A)** Increased T1 mapping. **(B)** Elevated T2 values of >55 milliseconds are indicative of edema.

Interval echocardiography showed improved left ventricular ejection fraction from 20% to 45%. Cardiac magnetic resonance imaging was significant for late gadolinium enhancement (LGE) at the basal inferoseptal segment of the right ventricular septum in a nonischemic pattern (Figure 3). Additional tissue characterization was performed at the segments without LGE. There was increased T1 mapping (seen in inflammation and edema) and increased T2 mapping (specific for edema) at the basal anterior and anteroseptal segments with associated expansion of extracellular volume fraction 32% (normal 25% ± 4%) (Figure 4).

His hospitalization after extubation was uneventful. He was given instructions to restrict sports and weightlifting for at least 3 months and until he was cleared by his primary outpatient cardiologist.

## Discussion

Blunt chest injury (BCI) to athletes is relatively common; however, resulting life-threatening cardiac conditions are rare. Cardiac injuries include pericardial effusion/tamponade, myocardial rupture, septal/valvular injury, commotio cordis, and contusion leading to myocardial dysfunction.^1^ The mechanism of cardiac contusion in athletes is not well understood but it is possibly due to the high-velocity trauma causing structural changes to the myocardium.^1^ It can be difficult to determine whether the abnormality is a primary event (eg, an acute coronary syndrome that preceded trauma), a direct result of cardiac injury, or a problem brought on by the physiologic stress of severe trauma.^2^ Takotsubo cardiomyopathy has been reported after BCI and should be included in the differential diagnosis of heart failure in those with chest trauma.^3^

Initial assessment includes cardiac imaging and biomarkers. An ECG consistent with BCI may reveal persistent sinus tachycardia, a new bundle branch block, or ST-segment depressions/elevations.^4^ BCI, more often, does not lead to hemodynamic instability; thus, dedicated cardiac imaging is generally not necessary.^5^ However, an echocardiogram is typically performed in any patient with suspected BCI and persistent shock out of proportion to apparent injuries.^5^ Echocardiography is useful in trauma patients with signs of cardiac dysfunction to diagnose the cause of dysfunction and to rule out pericardial tamponade. Signs suggestive of injury include abnormal cardiac wall motion and decreased cardiac contractility.^5^ Troponin levels may be useful in the patient with BCI and ECG abnormalities. A meta-analysis of 28 studies involving 7,242 patients with blunt chest trauma showed high sensitivity and specificity in identifying myocardial contusion when combining cardiac biomarker measurements with electrocardiography.^2^ Based on a review of 10 prospective studies involving 1,609 patients with BCI, a normal high sensitivity troponin using a lower reference limit was associated with a high negative predictive value for clinically significant cardiac complications.^6^ The American College of Radiology Appropriateness Criteria for Blunt Chest Trauma–Suspected Cardiac Injury list chest CT or CT angiography with or with and without intravenous contrast as an appropriate study for the stable patient with suspicion of injury.^7^ There is little guidance for use of cardiac magnetic resonance imaging in this setting; however, given its high-resolution imaging and increased utility for diagnosis of myocardial injury, in our opinion it is an appropriate cardiac imaging test in those with suspected BCI.^8^

Initial management of patients with cardiac contusion is to stabilize hemodynamics and treat expectant heart failure, shock, and arrhythmias. Our patient had evidence of worsening heart failure with ECG showing ischemia and possibly injury. Coronary angiography ruled out dissection and coronary obstruction. Right heart catheterization supported the clinical presentation and findings of initial bedside echocardiography (ie, low output state with elevated filling pressures). Fortunately, the patient responded to diuresis and afterload reduction.

This case shows the importance of prompt diagnosis, management, and imaging of patients with cardiac contusion. Return-to-play guidelines in this patient population are not defined and there are very limited data to guide decisions. The goal for athletes with chest trauma is to return to play once treatment is completed, and each case is and dependent on the severity and stability of each athlete. We considered the same principles as those of myocarditis as both disease processes are transient, can cause heart failure, and produce significant arrhythmias. An ambulatory monitor, resting echocardiogram, and exercise ECG and 3- to 6-month exercise restriction are currently supported in the 2015 Task Force 3 on Eligibility and Disqualification Recommendations for Competitive Athletes.^9^ This is based on consensus data with limitations and stems from concerns of the effect of exercise on viral replication and risk of ventricular tachyarrhythmias.^10^ Our patient was monitored for a 3-month period away from sports to allow complete resolution of cardiac injury. The decision to return to play should be incorporated in a shared decision-making framework with the patient and could be considered sooner if there is normalization of ventricular function, absence of ventricular arrhythmias, and resolution of markers of myocardial inflammation.^9^

## Follow-Up

A 1-week ambulatory monitor did not reveal any significant arrhythmias and systolic function normalized on subsequent echocardiography.

## Conclusions

Cardiac contusion is a rare complication of chest trauma in athletes but can lead to significant myocardial dysfunction. Recognition as a potential etiology is required with early use of multimodality imaging and invasive management to accurately diagnose and treat cardiac contusion is needed. In most cases, medical therapy and observation is all that is necessary.

## Funding Support and Author Disclosures

The authors have reported that they have no relationships relevant to the contents of this paper to disclose.

## References

[1] Thomas R.D., De Luigi A.J. (2018). Chest trauma in athletes. Curr Sports Med Rep.

[2] Van Lieshout E.M.M., Verhofstad M.H.J., Van Silfhout D.J.T., Dubois E.A. (2021). Diagnostic approach for myocardial contusion: a retrospective evaluation of patient data and review of the literature. Eur J Trauma Emerg Surg.

[3] Ritchie D., Trott T., Bryant J., Stearley S., Adkins B. (2013). Takutsubo cardiomyopathy and flash pulmonary edema in a trauma patient. J Emerg Med.

[4] Sybrandy K.C., Cramer M.J., Burgersdijk C. (2003). Diagnosing cardiac contusion: old wisdom and new insights. Heart.

[5] Karalis D.G., Victor M.F., McAllister M.P. (1994). The role of echocardiography in blunt chest trauma: a transthoracic and transesophageal echocardiographic study. J Trauma.

[6] Guild C.S., deShazo M., Geraci S.A. (2014). Negative predictive value of cardiac troponin for predicting adverse cardiac events following blunt chest trauma. South Med J.

[7] Stojanovska J., Koweek L., Chung J. (2020). ACR appropriateness criteria blunt chest trauma-suspected cardiac injury. J Am Coll Radiol.

[8] Martinez M.W. (2015). Advanced imaging of athletes: added value of coronary computed tomography and cardiac magnetic resonance imaging. Clin Sports Med.

[9] Maron B.J., Udelson J.E., Bonow R. (2015). Eligibility and disqualification recommendations for competitive athletes with cardiovascular abnormalities: task force 3: hypertrophic cardiomyopathy, arrhythmogenic right ventricular cardiomyopathy and other cardiomyopathies, and myocarditis. Circulation.

[10] Patriki D., Baltensperger N., Berg J. (2021). A prospective pilot study to identify a myocarditis cohort who may safely resume sports activities 3 months after diagnosis. J Cardiovasc Trans Res.

